# Adjuvant Therapy for High-Risk Stage II Cutaneous Melanoma: Insights and Future Directions

**DOI:** 10.3390/cancers18111802

**Published:** 2026-06-01

**Authors:** Federico Pravisano, Jacopo Costa, Lorenzo De Marchi, Gaetano Pascoletti, Elena Poletto, Michele Del Vecchio, Andrea Spagnoletti, Carolina Cimminiello, Nikolaos Papadopoulos, Jacopo Pigozzo, Luisa Piccin, Gabriele Roccuzzo, Paolo Fava, Fabio Puglisi, Giuseppe Aprile, Alessandro Marco Minisini

**Affiliations:** 1 Department of Medical Oncology, Santa Maria della Misericordia Academic Hospital, Azienda Sanitaria Universitaria Friuli Centrale (ASUFC), 33100 Udine, Italy; federico.pravisano@asufc.sanita.fvg.it (F.P.); gaetano.pascoletti@asufc.sanita.fvg.it (G.P.); elena.poletto@asufc.sanita.fvg.it (E.P.); giuseppe.aprile@asufc.sanita.fvg.it (G.A.); 2 Department of Medical Oncology, AULSS4 Veneto Orientale, Portogruaro Hospital, 30036 Portogruaro, Italy; costa.jacopo@spes.uniud.it; 3 Department of Medicine (DMED), University of Udine, 33100 Udine, Italy; fabio.puglisi@uniud.it; 4 Unit of Melanoma Medical Oncology, Department of Medical Oncology, Fondazione IRCCS Istituto Nazionale dei Tumori, 20133 Milan, Italy; michele.delvecchio@istitutotumori.mi.it (M.D.V.); andrea.spagnoletti@istitutotumori.mi.it (A.S.); 5 Melanoma Unit, Division of Clinical and Experimental Oncology and Immunotherapy of Melanoma, European Institute of Oncology, 20141 Milan, Italy; carolina.cimminiello@ieo.it (C.C.); nikolaos.papadopoulos@ieo.it (N.P.); 6 Medical Oncology 2, Veneto Institute of Oncology IOV–IRCCS, 35128 Padua, Italy; jacopo.pigozzo@iov.veneto.it (J.P.); luisa.piccin@iov.veneto.it (L.P.); 7 Department of Medical Science, Section of Dermatology, University of Turin, 10124 Turin, Italy; gabriele.roccuzzo@unito.it (G.R.); paolo.fava@unito.it (P.F.); 8 Department of Medical Oncology, CRO di Aviano, National Cancer Institute, IRCCS, 33081 Aviano, Italy

**Keywords:** cutaneous melanoma, stage II melanoma, adjuvant therapy, immune checkpoint inhibitors, anti-PD-1 therapy, recurrence-free survival, circulating tumor DNA, gene expression profiling, prognostic biomarkers

## Abstract

The role of adjuvant therapy in stage III melanoma is well established in clinical practice. Because stage IIB–IIC melanoma carries a risk of recurrence and melanoma-specific mortality comparable to that of stage III disease, adjuvant approaches have also been developed for patients with thick stage II tumors. Both pembrolizumab and nivolumab have demonstrated efficacy in reducing the risk of local and distant recurrence in patients with high-risk stage II melanoma, whereas evidence supporting the use of BRAF-MEK inhibitors in this setting remains inconclusive. Combinations of immune checkpoint inhibitors, as well as immunotherapy combined with mRNA-based vaccines, are currently under investigation in clinical trials. However, given the non-trivial risk of immune-related adverse events, careful selection of patients with stage II disease who are most likely to derive meaningful benefit from adjuvant therapy is essential. In this context, several clinicopathologic variables and gene expression profiling-based prognostic tools have been proposed to refine risk stratification. To date, however, none of these instruments have been incorporated into routine clinical practice, and no validated predictive biomarkers are available. Accordingly, optimal patient selection for adjuvant therapy remains an important unmet clinical need in early-stage melanoma.

## Simple Summary

Adjuvant therapies for resected melanoma have evolved rapidly in recent years. In this review, we provide an up-to-date overview of current and emerging adjuvant treatment options for patients with high-risk, node-negative stage II melanoma. Particular emphasis is placed on the role of risk stratification in guiding therapeutic decision making. We discuss both clinicopathologic and genomic risk-assessment tools that may help distinguish between lower- and higher-risk stage II tumors. These approaches may assist clinicians in identifying patients most likely to benefit from adjuvant therapy, recognizing that the absolute benefit of treatment is greatest among those with a higher baseline risk of recurrence.

## 1. Introduction

Adjuvant therapy for high-risk melanoma was initially developed for patients with stage III disease. Large, prospective, randomized phase III trials have shown that both BRAF-MEK inhibitors (in patients with *BRAF*-mutant melanoma) and Programmed Death-Ligand 1 (PD-L1) inhibitors significantly prolong relapse-free survival (RFS) and distant metastasis-free survival (DMFS) when administered in the postoperative setting [1,2,3,4]. Notably, nivolumab is currently the only agent approved for use in resected stage IV melanoma. However, none of these pivotal trials have demonstrated a statistically significant improvement in overall survival (OS) as compared with placebo [5]. Although the benefit of adjuvant therapy in terms of RFS is well established for stage IIIB–IIID melanoma, its role in stage IIIA disease (defined by microscopic lymph node involvement) remains more controversial, particularly among patients with sentinel-node metastases measuring <1 mm, who were excluded from most pivotal studies.

According to the current American Joint Committee on Cancer (AJCC, 8th edition) staging system, stage II cutaneous melanoma includes tumors without regional lymph node or distant metastases (N0M0) and is subdivided into stage IIA (T2b–T3a), stage IIB (T3b–T4a), and stage IIC (T4b). Stage IIA disease is generally considered lower-risk, whereas stage IIB and IIC melanomas are classified as high-risk because of their greater tumor thickness and/or the presence of ulceration, both of which are associated with substantially increased recurrence rates and melanoma-specific mortality. Consistent with this observation, stage IIB/IIC melanoma may follow an aggressive clinical course: 10-year melanoma-specific survival (MSS) rates are 81% for T3b tumors, 83% for T4a tumors, and 75% for T4b tumors. Notably, 10-year MSS is lower for patients with stage IIB disease than for those with stage IIIA disease (82% vs. 88%) [6]. This apparent paradox may partly reflect the biological heterogeneity of melanoma and the limitations of an anatomy-based staging system. Although stage III disease is defined by nodal involvement, patients with stage IIIA melanoma may present with a relatively low nodal tumor burden and more favorable primary tumor characteristics. In addition, some patients classified as stage II may harbor occult micrometastatic disease not detected at diagnosis, potentially contributing to their unexpectedly poor outcomes. Disease recurrence within 5 years occurs in approximately 35% of patients with stage IIB disease and 43% of those with stage IIC disease [7]. Overall, stage II melanoma accounts for approximately 15% of melanoma diagnoses, with nearly half classified as stage IIB/IIC. Despite this proportion, stage IIB/IIC disease is estimated to account for up to 50% of melanoma-related deaths [8]. In light of this disproportionate burden and the prognostic overlap with stage III disease, increasing efforts have therefore been directed toward extending adjuvant treatment strategies to patients with high-risk stage II melanoma.

## 2. Immunotherapy in Stage II Melanoma: Current Clinical Practice

### 2.1. KEYNOTE 716

In the phase 3 KEYNOTE-716 trial, adjuvant pembrolizumab improved RFS and DMFS compared with placebo among 976 patients with completely resected stage IIB–IIC melanoma. Patients were randomly assigned in a 1:1 ratio to receive pembrolizumab 200 mg every 3 weeks for up to 17 cycles or placebo. After a median follow-up of 39.4 months, median RFS and DMFS were not reached in either group. The 3-year RFS rate was 76.2% in the pembrolizumab group and 63.4% in the placebo group (hazard ratio [HR] 0.62; 95% CI 0.49–0.79). The 3-year DMFS rate was 84.4% and 74.7%, respectively (HR 0.59; 95% CI 0.44–0.79). Although an earlier interim analysis suggested uncertain benefit in patients with stage IIC disease, this was not confirmed with longer follow-up. The absolute improvement in 3-year RFS was 13.2% for stage IIB tumors and 13.4% for stage IIC tumors; the corresponding absolute improvements in DMFS were 7.8% and 12.8% [9]. Adverse events (AEs) were more frequent in the pembrolizumab group, as expected. Grade 3–4 treatment-related AEs occurred in 17% of patients, and treatment discontinuation due to AEs occurred in 15.9%. The most common treatment-related AEs were pruritus (24.6%), fatigue (21.5%), diarrhea (18.6%), arthralgia (16.4%), and rash (16.1%). Immune-related AEs were reported in 37.9% of patients, most commonly thyroid dysfunction (27.8%), colitis (4.1%), skin reactions (3.1%), adrenal insufficiency (2.7%), hypophysitis (2.5%), and hepatitis (2.3%). In several cases, these toxicities required immunosuppressive therapy and/or long-term endocrine replacement [9]. Treatment benefit was consistent across subgroups defined by melanoma histopathologic subtype, mitotic rate, ulceration status, and the presence or absence of tumor-infiltrating lymphocytes [10]. A recent analysis of the study evaluated whether clinical outcomes differed according to primary tumor location. With a median follow-up of 39.4 months, pembrolizumab improved both RFS and DMFS across all sites: for head and neck, HR 0.60 (95% CI 0.38–0.93) and 0.65 (95% CI 0.37–1.14), respectively; for trunk, 0.57 (95% CI 0.38–0.84) and 0.59 (95% CI 0.38–0.92); and for extremities, 0.53 (95% CI 0.31–0.90) for both endpoints [11]. Analysis of *BRAF* mutational status was not mandatory during screening, and it remains unknown whether outcomes differ between patients with *BRAF*-mutated and *BRAF* wild-type tumors [12]. Results were recently updated with a median follow-up of 52.8 months. The benefit of pembrolizumab in terms of RFS and DMFS was maintained, with 4-year RFS of 71% in the experimental arm versus 58% in the placebo arm (HR 0.62; 95% CI 0.50–0.78) and 4-year DMFS of 81% versus 70% (HR 0.59; 95% CI 0.45–0.77). Based on these results, the number needed to treat to prevent one recurrence was approximately 8. No new safety signals were identified [13]. Seventy-one patients in the placebo arm (41 with resectable disease and 30 with unresectable disease) crossed over to pembrolizumab after recurrence. Among the 41 patients with resectable disease, 17 (41%) experienced a further relapse (median RFS not reached). Among the 30 patients with unresectable disease, the objective response rate (ORR) was 43%, and median progression-free survival was 22 months [13].

These findings supported the approval of pembrolizumab by the U.S. Food and Drug Administration (FDA) and the European Medicines Agency (EMA) for adjuvant treatment of stage IIB–IIC resected melanoma.

### 2.2. CHECKMATE-76K

The phase 3, randomized, double-blind, placebo-controlled CHECKMATE-76K trial investigated adjuvant nivolumab administered every 4 weeks for up to 12 months in 790 patients with completely resected stage IIB/C melanoma. Patients were randomized to nivolumab or placebo, with RFS as the primary endpoint; secondary endpoints included DMFS, OS, PFS2, and safety. Approximately 60% of patients in both arms had stage IIB disease and 40% had stage IIC. About 50% of melanomas were nodular, 37% were located on the trunk in the nivolumab arm (34% in the placebo arm), and most tumors were *BRAF* wild-type (56% and 52%, respectively). PD-L1 status was unknown in approximately 60% of patients [14]. Treatment-related AEs of grade 3 or higher occurred in approximately 10% of patients receiving nivolumab, and 15% discontinued therapy due to treatment-related AEs; one treatment-related death was reported. Immune-related AEs included thyroid disorders (19%), skin rash (9%), colitis (5%), hepatitis (4%), and adrenal insufficiency (2%) [14]. With a median follow-up of 34.2 months for nivolumab and 33.9 months for placebo, 3-year RFS was 71% versus 61% (HR 0.62; 95% CI 0.47–0.80), and 3-year DMFS was 79% versus 74% (HR 0.72; 95% CI 0.52–1.00). Benefit was observed across all subgroups and was independent of disease stage (HR 0.59 for stage IIB, 0.65 for stage IIC), T category (HR 0.59 for T3b, 0.65 for T4b), primary tumor location (with slightly greater benefit in tumors of the arm and head/neck), and melanoma histopathologic subtype (greater benefit in nodular melanoma) [15]. Exploratory biomarker analyses suggested that high tumor mutational burden, elevated interferon gamma score, and CD8 T cell infiltration were associated with greater RFS benefit, whereas tumor mitotic rate and PD-L1 expression were not predictive [16]. With extended follow-up (median follow-up 46.8 months for nivolumab and 46.4 months for placebo), adjuvant nivolumab continued to demonstrate clinically meaningful reductions in the risk of recurrence and distant metastasis. At 4 years, Kaplan–Meier estimates of RFS were 66.1% in the nivolumab arm compared with 55.3% in the placebo arm (HR 0.64; 95% CI 0.50–0.82), and DMFS was 74.5% versus 66.8% (HR 0.73; 95% CI 0.54–0.98). Among 526 patients assigned to nivolumab and 264 to placebo, recurrence occurred in 131 (24.9%) and 100 (37.9%) patients, with distant recurrence in 65 (12.4%) and 52 (19.7%). The most frequent sites of distant relapse were lung (9.5% vs. 14.0%), lymph node (4.4% vs. 8.7%), liver (3.4% vs. 3.4%), and brain (1.9% vs. 4.5%). The treatment benefit was maintained beyond first progression, with median PFS2 not reached (HR 0.76; 95% CI 0.54–1.08), indicating continued disease control following subsequent therapy [17].

Evidence from the CHECKMATE-76K study led the FDA and EMA to approve nivolumab for the adjuvant treatment of stage IIB and IIC resected melanoma. Updated results from KEYNOTE 716 and CHECKMATE-76K are shown in Table 1.

## 3. Immunotherapy in Stage II Melanoma: Future Perspectives

### 3.1. Combination Immunotherapy Strategies in the Adjuvant Setting

The most promising recent innovation in adjuvant melanoma therapy probably comes from the phase 2b KEYNOTE-942 trial which tested an mRNA vaccine (V940) encoding 34 tumor-specific neoantigens designed to sensitize the patient’s immune system against melanoma cells. The trial enrolled 157 patients who were randomized 2:1 to receive V940 plus pembrolizumab or pembrolizumab alone. The combination significantly prolonged RFS in patients with stage IIIB–IV NED melanoma compared with pembrolizumab monotherapy (HR 0.56; 95% CI 0.309–1.17), with increased separation of the curves noted after approximately 40 weeks, suggesting a delayed effect. DMFS, tested hierarchically following the positive RFS result, was also longer in the combination arm (HR 0.347; 95% CI 0.145–0.828). Benefit was observed irrespective of TMB and PD-L1 expression [18]. At the 3-year update presented at ASCO 2024, the combination continued to demonstrate improved outcomes, with HRs of 0.51 (95% CI 0.288–0.906) for RFS and 0.384 (95% CI 0.172–0.858) for DMFS [19]. No new safety signals were identified. Although the mechanism of action of the mRNA vaccine is not fully elucidated, it is hypothesized to broaden antitumor responses and enhance the immune effects of pembrolizumab. The safety profile was reassuring: most AEs were grade 1–2, predominantly flu-like symptoms and local injection-site reactions, and no increase in immune-related AEs was observed with the addition of the vaccine [19]. The combination of V940 and pembrolizumab is now being evaluated in the phase 3 V940-001 trial (NCT05933577) among patients with resected high-risk (stage IIB–IV) melanoma. Primary endpoint is RFS, and secondary endpoints include DMFS, OS, safety, and quality of life [20].

T cell immunoglobulin and ITIM domain (TIGIT) and PD-1 are frequently co-expressed on tumor antigen-specific CD8^+^ T cells and CD8^+^ tumor-infiltrating lymphocytes (TILs). Anti-TIGIT antibodies block the interaction between TIGIT and its ligands (CD112 and CD155), thereby enhancing T cell activation against tumor cells. Preclinical studies suggest that combining anti-PD-1/PD-L1 with anti-TIGIT antibodies promotes proliferation and cytotoxic activity of CD8^+^ T cells [21]. The anti-TIGIT antibody vibostolimab has demonstrated activity against solid malignancies in a phase 1 trial, both as monotherapy and in combination with pembrolizumab, with an acceptable safety profile. Although no melanoma patients were included, the trial provides important preliminary data. In part B, among 106 patients with non-small cell lung cancer, 34 immune checkpoint inhibitor (ICI)-refractory patients received vibostolimab monotherapy, and 33 ICI-refractory patients received the combination. Objective response rates were 3% in both arms, whereas disease control rates were 32% with monotherapy and 40% with the combination. Six-month PFS rates were 10% and 25%, respectively, and median OS was 11 months with monotherapy versus 13 months with the combination. Treatment was generally well tolerated, with the most common AEs being fever and hypoalbuminemia. Despite the small sample size and exploratory nature, these results are encouraging regarding antitumor activity [22]. A co-formulation of pembrolizumab and vibostolimab, administered every 3 weeks for up to 17 cycles, was evaluated as adjuvant therapy for resected stage IIB–IV melanoma in the phase 3, double-blind KEYVIBE-010 trial, compared with pembrolizumab alone. The trial was prematurely discontinued due to a high incidence of immune-related AEs in the experimental arm [23].

In an effort to enhance the antitumor activity of anti-PD-1 therapy, combinations of lymphocyte activation gene 3 (LAG-3) inhibitors with anti-PD-1 monoclonal antibodies have been investigated in advanced melanoma, showing promising results. In particular, the combination of relatlimab (anti-LAG-3) and nivolumab improved PFS, OS, and ORR compared with nivolumab alone in first-line treatment of metastatic melanoma, with a safety profile largely comparable to single-agent anti-PD-1 therapy. The benefit of the combination was maintained with a median follow-up of 33.8 months [24]. This combination was subsequently evaluated versus nivolumab alone in the adjuvant setting for patients with resected stage III–IV melanoma in the randomized, double-blind RELATIVITY-098 trial. The study did not meet its primary endpoint, and no significant improvement in RFS was observed [25]. One possible explanation is that patients with completely resected melanoma may lack a sufficient pool of antitumor T cells to be effectively activated by LAG-3 inhibition, in contrast to the neoadjuvant setting, where the same combination has demonstrated clinical activity [26]. For patients with stage IIB–IV resected melanoma, adjuvant therapy with the LAG-3 inhibitor fianlimab plus the anti-PD-1 cemiplimab is currently under investigation versus pembrolizumab alone in the phase 3, double-blind R3767-ONC-2055 trial [27].

### 3.2. Immune Checkpoint Blockade in the Neoadjuvant Setting

The NADINA trial demonstrated a marked superiority of neoadjuvant, response-driven adjuvant combination immunotherapy compared with adjuvant immunotherapy alone in patients with macroscopic stage III melanoma, defined as clinically positive regional lymph nodes [28]. Similarly, pembrolizumab administered in a perioperative schedule showed improved outcomes compared with adjuvant therapy alone in stage IIIB–IV resectable melanoma in a randomized phase II SWOG trial [29]. Although data for neoadjuvant therapy in stage II melanoma are still limited, several early-phase studies are exploring this approach.

In the randomized, double-blind, placebo-controlled phase II INTRIM trial, in patients with resectable pT3–T4 cN0 melanoma, a single intradermal administration of the Toll-like receptor 9 (TLR9) agonist tilsotolimod at the primary tumor excision site 7–10 days prior to surgery significantly reduced the rate of tumor-positive sentinel lymph nodes compared with placebo (13% vs. 45%). At a median follow-up of 24 months, tilsotolimod was associated with a marked improvement in RFS (HR 0.31; 95% CI 0.14–0.77); the incidence of distant recurrence was also reduced (6.4% vs. 19.4%), supporting a potential systemic immune effect following local intralesional TLR9 activation [30].

The single-country phase 1b/2 MARIANE trial is investigating whether intradermal injection of ipilimumab plus nivolumab at the primary tumor excision site can induce a local antitumor response in high-risk (T3–T4) stage II patients [31].

In addition, the phase 2 NeoReNi II trial is evaluating neoadjuvant nivolumab in combination with relatlimab in 20 patients with stage II melanoma, with the primary objective of estimating pathological response rates after two cycles of therapy [32].

Furthermore, the phase 2 NCT03757689 trial assessed the effect of a single preoperative dose of pembrolizumab administered 3 weeks before wide excision and sentinel lymph node biopsy in 63 patients with clinical stage IIB/IIC melanoma. All patients subsequently received standard adjuvant pembrolizumab for one year. The primary endpoint was the rate of sentinel node positivity compared with historical treatment-naive controls (estimated at 33.1%). Although perioperative pembrolizumab reduced sentinel node positivity by 18% overall, the reduction did not reach statistical significance. However, in the stage IIC subgroup, sentinel node positivity was significantly lower (23.5% vs. 40%). No new safety signals were reported, and surgery was not delayed [33].

Ongoing trials investigating neoadjuvant immune-based therapies in stage II melanoma, including both actively recruiting studies and studies closed to enrollment but awaiting adequate follow-up, are shown in Table 2.

## 4. BRAF and MEK Inhibitors in the Adjuvant Treatment of Stage II *BRAF*-Mutant Melanoma: An Undefined Role

Limited data are available on the efficacy of BRAF and MEK inhibitors as adjuvant therapy in stage II melanoma. The phase 3, international, placebo-controlled, randomized BRIM 8 trial evaluated the BRAF inhibitor vemurafenib in patients with resected stage IIC-III *BRAF V600*-mutant melanoma. Adjuvant treatment was administered for 52 weeks. Cohort 1 included 314 patients with stage IIC-IIIB disease, while cohort 2 enrolled 184 patients with stage IIIC disease. The primary endpoint was DFS, with a hierarchical analysis planned (cohort 2 before cohort 1). Median follow-up was 33.5 months in cohort 2 and 30.8 months in cohort 1. Although a statistically significant DFS benefit was observed in cohort 1 (HR 0.54; 95% CI 0.37–0.78), the trial was considered negative overall, as the primary endpoint was not met in cohort 2 (HR 0.80; 95% CI 0.54–1.18). Grade 3–4 AEs occurred in 57% of patients in the experimental arm [34]. It should be noted that single-agent BRAF inhibition is no longer standard of care, as combination therapy with a MEK inhibitor is now recommended unless contraindications exist, such as ocular or cardiac disease [35]. The phase 3 COLUMBUS-AD trial, designed to investigate adjuvant BRAF/MEK inhibition in high-risk stage II *BRAF*-mutated melanoma, was prematurely closed due to slow accrual [36]. In this international, randomized, placebo-controlled, triple-blind study, patients with resected stage IIB-IIC *BRAF V600E/K*-mutant melanoma were randomized 1:1 to receive adjuvant encorafenib plus binimetinib or placebo for 12 months. The primary endpoint was RFS. Early termination was largely due to the challenges of maintaining a placebo-controlled design after the approval of adjuvant immunotherapy for stage IIB/IIC melanoma. Among the 110 patients recruited (65% stage IIB, 35% stage IIC), safety and RFS data were recently presented. In the experimental arm, grade ≥3 AEs occurred in 24% of patients, and 33% discontinued treatment due to an AE. Descriptive 1-year RFS was 86% (95% CI 65–95%) with encorafenib/binimetinib versus 70% (46–85%) with placebo; 1-year DMFS was 92% (77–97%) versus 82% (55–93%), respectively [37]. To date, to the best of our knowledge, no other ongoing trials are investigating BRAF and MEK inhibitors as adjuvant therapy for high-risk stage II melanoma.

## 5. Identifying the Optimal Candidate for Adjuvant Therapy in Stage II Melanoma

It is widely accepted that adjuvant anti-PD-1 therapy should be offered to patients with high-risk stage II melanoma. However, the optimal selection of patients who are most likely to benefit from this approach remains an unmet clinical need. Several biomarkers including PD-L1 expression, mismatch repair status, LAG-3 expression, T cells at tumor invasive margins, MHC, and IL-17 expression, have largely failed to predict response to immunotherapy in early-stage and advanced melanoma [38].

### 5.1. Clinicopathological Prognostic Models

The Melanoma Institute of Australia developed a multivariable prognostic model to estimate individualized 5- and 10-year RFS and OS in stage II melanoma. The model incorporates clinical variables (age, sex) and pathological features (Breslow thickness, tumor proliferative rate, TILs, regression, lymphovascular invasion, anatomical site, satellite lesions, melanoma histopathologic subtype). Validated for sentinel-node-negative patients and those without sentinel node biopsy, this model may guide selection of patients likely to derive the greatest absolute RFS benefit from adjuvant therapy [39].

Dixon et al. proposed the BAUSSS prognostic algorithm, which integrates Breslow thickness, age, ulceration, melanoma subtype, sex, and anatomical site to improve survival prediction in patients with localized cutaneous melanoma without clinical evidence of nodal or distant metastases. The authors suggested that this clinicopathological model may provide more accurate individualized prognostic stratification than AJCC staging alone and could help identify patients at sufficiently high risk to be considered for adjuvant systemic therapy. Notably, the BAUSSS model was designed as a non-invasive prognostic tool that does not rely on sentinel lymph node biopsy, highlighting the potential role of clinicopathological algorithms in refining risk assessment while reducing procedure-related morbidity and costs [40].

High levels of TILs also appear to be a favorable prognostic biomarker in early-stage melanoma, although prospective validation is required before routine use [41,42]. Preliminary data suggest that certain metabolic conditions, including type 2 diabetes mellitus, may be associated with poorer RFS in patients with stage II–III melanoma receiving adjuvant anti-PD-1 therapy [43]. Chronic hyperglycemia and insulin resistance may promote an immunosuppressive tumor microenvironment and dysregulated immune responses, potentially reducing the efficacy of PD-1 blockade [44].

### 5.2. Circulating Tumor DNA-Guided Strategies

Circulating tumor DNA (ctDNA) detection after melanoma resection appears to correlate with a higher risk of recurrence in stage II–III disease. Most patients with detectable ctDNA within 12 weeks of curative-intent surgery relapse within one year, and ctDNA may uncover metastatic disease not evident on imaging [45]. In a recent retrospective cohort study, Steimle et al. evaluated a personalized, tumor-informed, exome-based ctDNA assay in patients with resected stage II–IV melanoma undergoing surveillance and found an overall sensitivity of 53.6% for detecting recurrence, with performance strongly influenced by site of relapse, ranging from approximately 87% for nodal and liver metastases to 33–38% for brain and cutaneous disease, highlighting the assay’s prognostic value but its limited reliability as a stand-alone surveillance tool [46]. Although ctDNA may provide prognostic information to identify patients at higher risk of recurrence, its utility for guiding adjuvant therapy remains unproven. The DETECTION trial was designed to evaluate whether postoperative ctDNA could guide adjuvant therapy in stage IIB/IIC melanoma, potentially sparing unnecessary treatment. Patients with detectable ctDNA were randomized 1:1 to receive adjuvant nivolumab or continued surveillance with ctDNA monitoring, routine scans, and investigator-selected therapy upon recurrence. The trial was prematurely closed due to changes in standard-of-care guidelines, which rendered surveillance alone unethical in high-risk stage II patients [47]. To further explore a risk-adapted strategy based on molecular relapse, the ongoing DETECTION-2 feasibility study is assessing the practicality of a ctDNA-guided approach in patients with resected stage IIB/IIC/IIIA melanoma. In this study, patients with *BRAF*, *NRAS*, or *TERT* promoter-mutant tumors are randomized 1:1 to standard adjuvant therapy or serial ctDNA monitoring, with treatment initiated only upon ctDNA detection. Tumor-informed droplet digital PCR assays are performed at regular intervals (every 3 months for 3 years, then every 6 months up to year 5). The primary objectives are to evaluate recruitment feasibility and turnaround times for ctDNA testing to inform the design of a future phase 3 trial comparing ctDNA-guided early intervention with routine adjuvant therapy [48].

### 5.3. Gene-Expression Profiling and Molecular Risk Stratification

The ongoing phase 3 NIVOMELA trial (NCT04309409) uses MELAGENIX, a quantitative RT-PCR-based 11-gene expression assay (eight prognostic and three reference genes), to stratify relapse risk and guide adjuvant treatment decisions in stage II melanoma [49]. The gene expression profiling (GEP) score derived from the MELAGENIX assay has been validated in 245 patients by Amaral et al., with scores ranging from −0.7 to 3.53. At a median follow-up of 40 months, low-risk tumors (GEP ≤ 0) had markedly better MSS at 5 and 10 years (92%) than high-risk tumors (GEP > 0; 5-year MSS 82%, 10-year MSS 67%) [50]. In NIVOMELA, patients with low-risk tumors undergo observation without adjuvant therapy, whereas those with high-risk tumors are randomized to adjuvant nivolumab or placebo.

A German validation study combined clinicopathological features (age at diagnosis, Breslow thickness) with expression of eight tumor genes (*PLAT*, *MLANA*, *LOXL4*, *SERPINE2*, *ITGB3*, *GDF15*, *TGFBR1*, *CXCL8*) to develop a prognostic score in 543 stage I/II patients with negative sentinel node biopsy. Median follow-up was 83.6 months. Five-year RFS for high-risk stage I–IIA tumors resembled that of AJCC 8th edition stage IIB/C tumors (77.8%), suggesting this score could help identify early-stage melanomas warranting adjuvant therapy [51].

Additionally, the 31-gene expression profile (31-GEP; DecisionDx-Melanoma) prospectively predicts MSS and OS in early-stage melanoma [52]. Patients with class 1A results have significantly better MSS and OS than those with class 2B. Retrospective analysis of 1662 stage I–II patients suggests that 31-GEP can stratify risk of central nervous system (CNS) metastases, with class 2B patients at higher risk, potentially guiding CNS imaging [53].

Sánchez-Beltrán et al. identified a simple 4-gene expression signature based on *MGRN1*, *MLANA*, *PMEL*, and *TYRP1* that improved prognostic stratification beyond conventional TNM staging, particularly among patients with low- to intermediate-stage melanoma. Notably, this signature identified a subgroup of clinically low-risk patients with unexpectedly poor outcomes and was associated with dysregulation of inflammatory pathways, cell-cycle progression, and DNA damage/repair programs, supporting the biological heterogeneity underlying stage II melanoma behavior [54].

Garg et al. developed the Cam_121 gene expression signature using transcriptomic data from patients with AJCC stage IIB/IIC and stage III melanoma enrolled in the AVAST-M trial. The signature improved prediction of metastasis and survival beyond conventional clinicopathological variables and identified a subgroup of stage II patients with markedly increased risk of death and an immune-depleted tumor microenvironment, supporting its potential role in refining selection for adjuvant therapy [55].

More recently, van den Hurk et al. identified *LY75* promoter methylation as an independent predictor of recurrence and metastatic progression in localized stage I/II melanoma. Importantly, this epigenetic marker provided prognostic information beyond conventional clinicopathological variables and was proposed as a potential tool to refine selection of patients who may benefit from intensified surveillance, sentinel lymph node biopsy, or (neo)adjuvant treatment strategies [56].

It should be highlighted that, although some of the emerging clinicopathological and molecular/epigenetic models discussed above may eventually help identify patients in whom sentinel lymph node biopsy (SLNB) could potentially be omitted, SLNB currently remains an important component of risk stratification in high-risk stage II melanoma. Beyond its prognostic value, detection of occult nodal involvement may upstage patients to stage III disease and allow consideration of adjuvant targeted therapy in *BRAF*-mutated melanoma, an option that avoids some of the potentially permanent immune-related toxicities associated with anti-PD-1 therapy [57].

Ongoing trials of adjuvant therapy in stage II melanoma, including actively recruiting studies and studies closed to enrollment but awaiting adequate follow-up, are shown in Table 3.

## 6. Conclusions

Stage IIB/IIC melanoma represents a heterogeneous disease and carries a worse prognosis than stage IIIA. Both pembrolizumab and nivolumab are approved by the FDA and EMA as adjuvant therapies for resected stage IIB/IIC melanoma, having demonstrated reductions in the risk of recurrence compared with placebo. Pembrolizumab increases 4-year RFS by approximately 13% and 4-year DMFS by about 11%. Notably, 58% of patients remain relapse-free at 4 years without adjuvant therapy, highlighting a potentially high rate of overtreatment. Adjuvant pembrolizumab is associated with a 17% incidence of grade 3 treatment-related AEs, some of which may result in permanent endocrine dysfunction. Adjuvant nivolumab has shown similar efficacy and safety, increasing 4-year RFS by roughly 11% and 4-year DMFS by about 7%. Overall survival benefit has not yet been demonstrated for high-risk stage II melanoma treated with adjuvant immunotherapy. Current research in stage II melanoma focuses on two main objectives.

First, the development of refined prognostic tools—including gene expression profiling assays, epigenetic models, postoperative ctDNA detection, and clinicopathological scores—aims to identify patients at higher risk of relapse who may derive the greatest absolute benefit from adjuvant therapy, while sparing low-risk patients from unnecessary toxicity and potentially avoiding invasive staging procedures such as sentinel lymph node biopsy.

Second, novel therapeutic combinations—including dual immune checkpoint blockade or ICIs combined with mRNA vaccines—are being investigated to further reduce the risk of recurrence while maintaining an acceptable safety profile and preserving quality of life. Of note, several studies are exploring neoadjuvant ICI-based approaches in high-risk stage II melanoma, an approach already showing practice-changing results in clinically node-positive stage III disease.

Finally, even in patients not selected for (neo)adjuvant systemic therapy, careful long-term surveillance remains essential, including regular dermatologic examinations, nodal assessment and radiologic monitoring according to recurrence risk and guideline recommendations, in order to enable early detection of disease relapse [35].

## Figures and Tables

**Table 1:** Updated results from KEYNOTE 716 and CHECKMATE-76K trials.

Trial	Median FU (Months)	Relative RFS Benefit	Absolute RFS Benefit	NNT for RFS Benefit
KEYNOTE 716 [13] 4-years update	52.8 m	38% (HR 0.62)	+13%	8
CHECKMATE-76K [17] 4-years update	46.8 m	36% (HR 0.64)	+11%	9

FU: Follow-up; RFS: Relapse-free survival; NNT: Number needed to treat; and HR: Hazard ratio.

**Table 2:** Ongoing trials of neoadjuvant immunotherapy in stage II melanoma.

Trial	Phase	Disease Stage	Intervention Drugs	Adjuvant Therapy	Primary Endpoints	Secondary Endpoints
INTRIM-1 [30]	2	pT3Nx or T4Nx primary cutaneous melanoma	Intradermal single injection of tilsotolimod (8 mg) at the primary melanoma excision site one week before SLN biopsy	Standard of care anti-PD-1 therapy in patients with stage III disease	SLN positivity rate	RFS OS Immune response in the SLN and peripheral blood
MARIANE [31]	1b/2	pT3-4 melanoma with ≥ 44% risk for SLN positivity using MIA Sentinel Node Metastasis Risk prediction tool	Phase 1b: intradermal ipi + nivo at different schedules and doses Phase 2: Optimal intradermal ipi + nivo regimen vs. 2 cycles of intravenous nivo	Standard of care anti-PD-1 in patients with a non-MPR (RCB > 10% of vital cells)	Pathological response Feasibility	TRAEs EFS DMFS OS
NeoReNi [32]	2	IIA (T2b, T3a), IIB (T3b, T4a) or IIC (T4b) primary cutaneous melanoma	Neoadjuvant treatment with 2 cycles of nivolumab (480 mg, IV) + rela (160 mg, IV) q28	Further 11 cycles of adjuvant nivo (480 mg) + rela (160 mg) in patients with a non-MPR (RCB > 10% of vital cells)	Pathological response Feasibility	SLN positivity rate EFS RFS OS Safety of neoadjuvant and adjuvant therapy PROs
NCT03757689 [33]	2	IIB (T3b, T4a) or IIC (T4b) primary cutaneous melanoma	Neoadjuvant treatment with one dose of pembrolizumab 200 mg followed by wide excision and SLN biopsy	Standard of care anti-PD-1 therapy with pembrolizumab	SLN positivity rate	DFS OS

DMFS: distant metastasis-free survival; EFS: event-free survival; ipi: ipilimumab; nivo: nivolumab; rela: relatlimab; OS: overall survival; PROs: patient-reported outcomes; RCB: residual cancer burden; RFS: relapse-free survival; SLN: sentinel lymph node; MPR: major pathologic response; TRAEs: treatment-related adverse events.

**Table 3:** Ongoing clinical trials of adjuvant therapy in stage II melanoma.

Trial	Phase	Disease Stage	Intervention Drugs	Primary Endpoints	Key Secondary Endpoints
R3767-ONC-2055 [27]	3	IIB–IV NED	1-year therapy with fianlimab (HD or LD) + cemiplimab vs pembrolizumab	RFS	DMFS OS TRAEs, including irAEs PROs
V940-001 [18,19]	3	IIB–IV NED	1-year therapy with mRNA-4157 (q3W, up to 9 doses) + pembrolizumab 400 mg vs placebo + pembrolizumab 400 mg	RFS	DMFS OS AEs QoL
NIVOMELA [50]	3	IIA–IIC	If MelaGenix GEP score > 0.0 (high risk of relapse) 1-year therapy with nivolumab 480 mg vs. observation	RFS	DFS OS MSS AEs > G3 Clinical utility of the MelaGenix GEP score in stratifying patients
DETECTION-2 [48]	FT	IIB–IIIA	Standard therapy with anti-PD-1 vs ctDNA monitoring with initiation of therapy upon molecular relapse	Recruitment rate in 12 months Proportion of ctDNA results returned to site ≤10 working days	RFS DMFS OS Percentage of clinical/radiological relapses with negative ctDNA

FT: Feasibility trial; NED: no evidence of disease; HD: high dose; LD: low dose; ctDNA: circulating tumor DNA; RFS: relapse-free survival; DMFS: distant metastasis-free survival; OS: overall survival; TRAEs: treatment-related adverse events; irAEs: immune-related adverse events; PROs: patient-reported outcomes; QoL: quality of life; GEP: gene expression profile; and MSS: melanoma-specific survival.

## Data Availability

No new data were created in this study. Data sharing is not applicable to this article.
